# Malaria elimination in Malaysia and the rising threat of *Plasmodium knowlesi*

**DOI:** 10.1186/s40101-020-00247-5

**Published:** 2020-11-23

**Authors:** Abraham Zefong Chin, Marilyn Charlene Montini Maluda, Jenarun Jelip, Muhammad Saffree Bin Jeffree, Richard Culleton, Kamruddin Ahmed

**Affiliations:** 1 Department of Community and Family Medicine, Faculty of Medicine and Health Sciences, Universiti Malaysia Sabah, 88400 Kota Kinabalu, Sabah Malaysia; 2 Disease Control Division, Ministry of Health, 62590 Putrajaya, Selangor Malaysia; 3 Borneo Medical and Health Research Centre, Universiti Malaysia Sabah, 88400 Kota Kinabalu, Sabah Malaysia; 4 Division of Molecular Parasitology, Proteo-Science Center, Ehime University, Toon, Ehime 791-0295 Japan; 5 Department of Pathobiology and Medical Diagnostics, Faculty of Medicine and Health Sciences, Universiti Malaysia Sabah, Jalan UMS, 88400 Kota Kinabalu, Sabah Malaysia

**Keywords:** Malaria, Malaysia, Epidemiology, Zoonotic malaria, Elimination

## Abstract

**Background:**

Malaria is a major public-health problem, with over 40% of the world’s population (more than 3.3 billion people) at risk from the disease. Malaysia has committed to eliminate indigenous human malaria transmission by 2020. The objective of this descriptive study is to understand the epidemiology of malaria in Malaysia from 2000 through 2018 and to highlight the threat posed by zoonotic malaria to the National Malaria Elimination Strategic Plan.

**Methods:**

Malaria is a notifiable infection in Malaysia. The data used in this study were extracted from the Disease Control Division, Ministry of Health Malaysia, contributed by the hospitals and health clinics throughout Malaysia. The population data used in this study was extracted from the Department of Statistics Malaysia. Data analyses were performed using Microsoft Excel. Data used for mapping are available at EPSG:4326 WGS84 CRS (Coordinate Reference System). Shapefile was obtained from igismap. Mapping and plotting of the map were performed using QGIS.

**Results:**

Between 2000 and 2007, human malaria contributed 100% of reported malaria and 18–46 deaths per year in Malaysia. Between 2008 and 2017, indigenous malaria cases decreased from 6071 to 85 (98.6% reduction), while during the same period, zoonotic *Plasmodium knowlesi* cases increased from 376 to 3614 cases (an 861% increase). The year 2018 marked the first year that Malaysia did not report any indigenous cases of malaria caused by human malaria parasites. However, there was an increasing trend of *P. knowlesi* cases, with a total of 4131 cases reported in that year. Although the increased incidence of *P. knowlesi* cases can be attributed to various factors including improved diagnostic capacity, reduction in human malaria cases, and increase in awareness of *P. knowlesi*, more than 50% of *P. knowlesi* cases were associated with agriculture and plantation activities, with a large remainder proportion linked to forest-related activities.

**Conclusions:**

Malaysia has entered the elimination phase of malaria control. Zoonotic malaria, however, is increasing exponentially and becoming a significant public health problem. Improved inter-sectoral collaboration is required in order to develop a more integrated effort to control zoonotic malaria. Local political commitment and the provision of technical support from the World Health Organization will help to create focused and concerted efforts towards ensuring the success of the National Malaria Elimination Strategic Plan.

## Introduction

Malaria is a major public health challenge, with over 40% of the world’s population (3.3 billion people) at risk from the disease. According to the latest World Malaria Report (2019), there were an estimated 228 million cases of malaria worldwide in 2018, with the majority of cases occurring in Africa.

Malaysia has come a long way in the prevention and control of malaria since the introduction of the Malaria Eradication Program in 1961 in Sabah and Sarawak, and in 1967 in Peninsular Malaysia [1]. In 2011, Malaysia recorded less than 1 case per 1000 people and thereby entered into the elimination phase of malaria control [2]. The National Malaria Elimination Strategic Plan was introduced in the same year with the target of achieving “malaria-free” status by 2020. Malaria-free status here refers to human indigenous malaria species such as *P. falciparum*, *P. malariae*, *P. ovale*, and *P. vivax.* In 2019, the WHO Western Pacific Region, which includes Malaysia, reported the second lowest malaria incidence among all WHO Regions [3]. Under the Regional Action Framework for Malaria Control and Elimination in the Western Pacific (2016–2020), three countries/provinces including Malaysia were targeted to achieve malaria elimination by 2020 [4].

*Plasmodium knowlesi* is a zoonotic malaria parasite transmitted between non-human primate hosts by *Anopheles* mosquitoes and causes spill-over infections in humans where the parasite, vector, host, and human converge. The first naturally acquired *knowlesi* malaria case was reported in 1965 in a man who returned to the USA after visiting peninsular Malaysia [5], followed by a second naturally occurring transmission reported in 1971 [6]. *Plasmodium knowlesi* is the fifth species that can cause malaria in humans and gained attention as a possible emerging public health threat in 2004 when a large focus of naturally acquired *P. knowlesi* infections was confirmed in blood samples from 2002 to 2004 in Sarawak, Malaysian Borneo [7]. Human *P. knowlesi* infections occurred in Sarawak from as early as 1996 [8], and also from 2000 to 2002 [7] and 2001 to2006 [9]. *P. knowlesi* cases were also described in Pahang in 2004 and 2005, and in Sabah from 2003 to 2005 [9].

Most human *P. knowlesi* cases are chronic and symptomatic, but some can be severe and lead to death [10]. Clinical studies in Sarawak indicated that more than 10% of patients with *P. knowlesi* malaria developed severe disease as classified by the WHO with an approximately 1% case fatality rate (CFR) [11]. *Plasmodium knowlesi* has the shortest asexual replication cycle of all human-infecting malaria parasites leading to rapidly increasing parasitemia levels [11]. It is highly sensitive to artemisinin, and variably and moderately sensitive to chloroquine and mefloquine [12].

Here, we analyze Malaysia’s successful anti-malaria campaign from an epidemiological perspective, with the aim that the results can be reference for other countries experiencing malaria elimination plan. The objective of this study is to understand the epidemiology of malaria in Malaysia from 2000 through 2018 and to highlight the threat that zoonotic malaria poses to the National Malaria Elimination Strategic Plan.

## Methods

Here, we present a descriptive study on the epidemiology of malaria in Malaysia from 2000 through 2018. The study site is whole Malaysia. The case definitions for classification of malaria are given in Table 1 [13]. Human malaria was defined as malaria caused by *P. falciparum*, *P. malariae*, *P. ovale*, and *P. vivax.* Only these four species of *Plasmodium* were only considered in indigenous malaria. Imported versus indigenous malaria cases were classified according to the criteria set by the WHO, which were consideration of travel history and incubation period, and the evidences of epidemiology link in traveled destination and the absence of epidemiology link locally during case investigation. The data used in this study were provided by the Disease Control Division, Ministry of Health Malaysia, and were initially contributed by hospitals and health clinics throughout Malaysia. Malaria is a mandatory notifiable disease under the Malaysian Infectious Disease Prevention and Control Act 1988. Passive surveillance is performed through routine notification of district health offices by local health facilities, while active surveillance among high-risk groups such as land scheme settlers and migrant workers is carried out in endemic areas. State health departments compile data for the whole state before sending it to the national level central health department. The population data used in this study was extracted from the Department of Statistics Malaysia. Data analyses were performed using Microsoft Excel. Data used for mapping are available at EPSG:4326 WGS84 CRS (Coordinate Reference System). Shapefile was obtained from igismap [14]. Mapping and plotting of the map were performed using QGIS [15].

**Table 1:** Case definition for the classification of malaria

Malaria case	Confirmed malaria case (or infection) in which the parasite has been detected in a diagnostic test, i.e., microscopy, a rapid test, or a molecular diagnostic test
Malaria case, introduced	A case contracted locally, with strong epidemiological evidence linking it directly to a known imported case (first-generation local transmission)
Malaria case, imported	Malaria case or infection in which the infection was acquired outside the area in which it is diagnosed
Malaria case, indigenous	A case contracted locally with no evidence of importation and no direct link to transmission from an imported case
Malaria elimination	Interruption of local transmission (reduction to zero incidence of indigenous case) of a specified (human) malaria parasite in a defined geographical area as a result of deliberate activities. Continued measures to prevent re-establishment of transmission are required

## Results

**Fig. 1:**
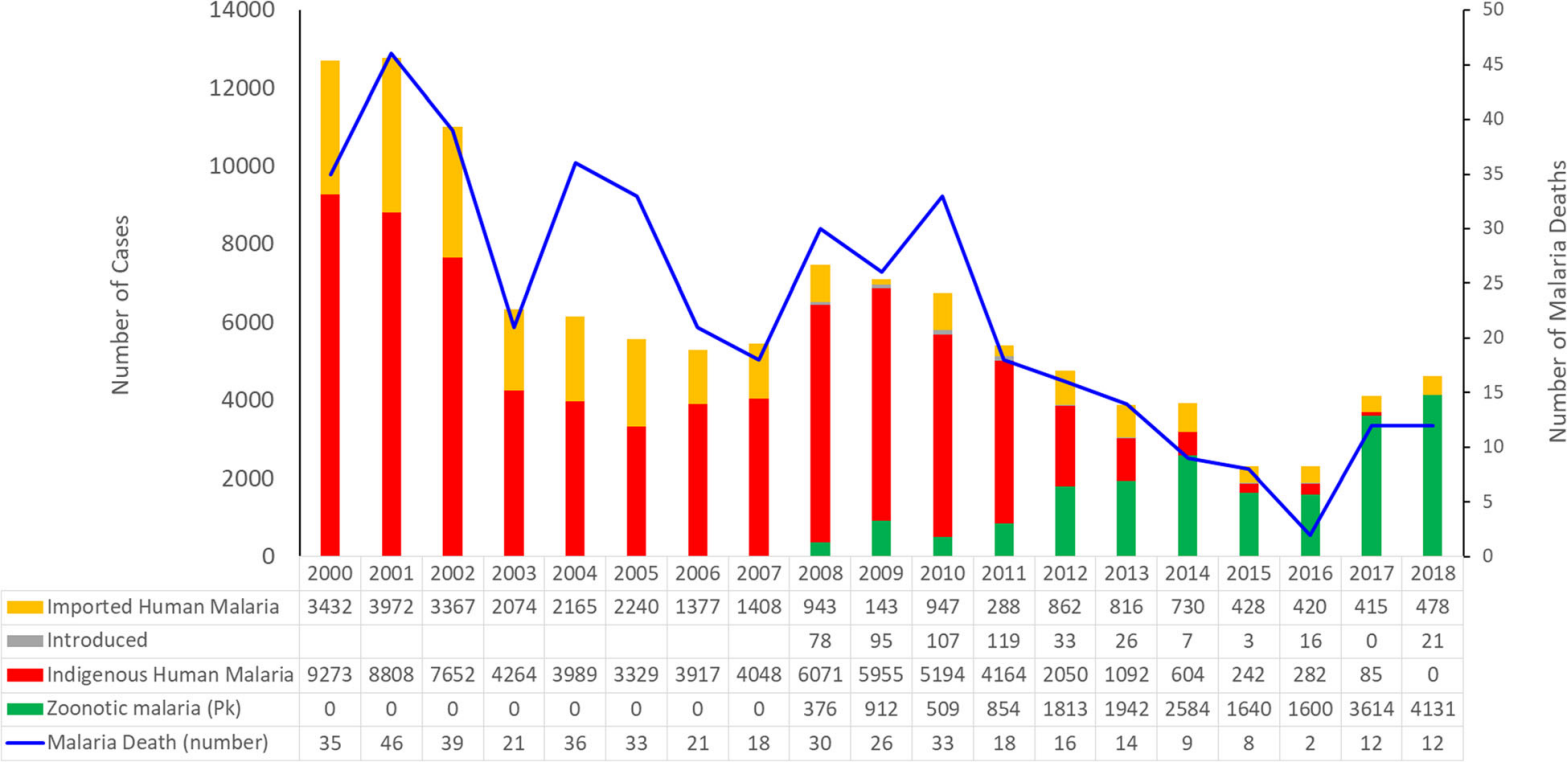
Annual reported malaria cases and deaths in Malaysia, 2000–2018

**Fig. 2:**
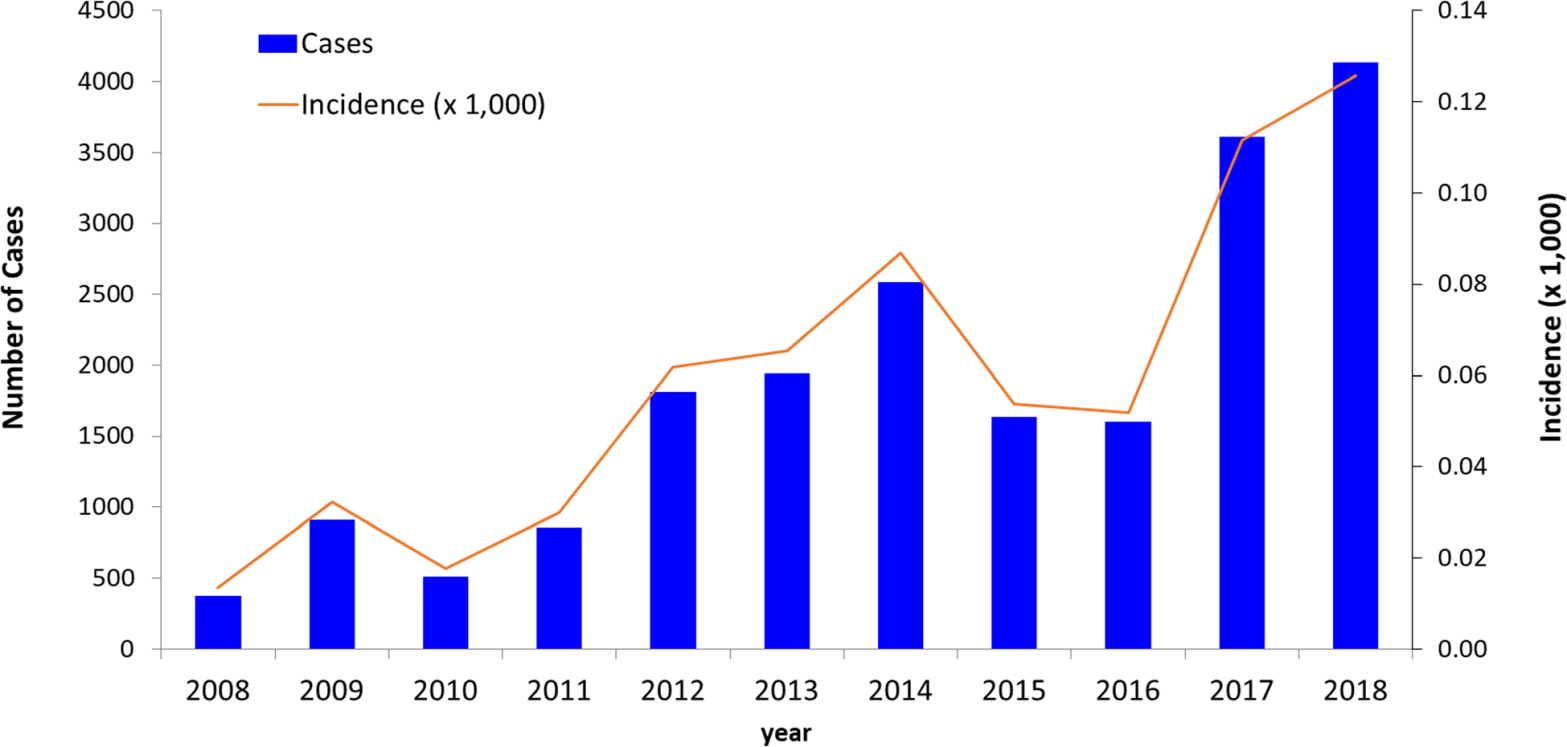
Incidence of *knowlesi* malaria in Malaysia, 2008–2018

The highest numbers of indigenous malaria cases (9273) were recorded in 2000. Cases decreased gradually to 3329 in 2005 (Fig. 1). There followed a gradual increase over the next 3 years to a peak of 6071 cases in 2008, followed by a further decrease to 2050 cases in 2012, and by 2018, no cases were detected. Converse to this trend, *Plasmodium knowlesi* malaria was first identified in 2008 (Figs. 1 and 2) and by 2018 accounted for a total of 4131 cases of malaria. Between 2000 and 2007, human malaria contributed to 100% of reported malaria cases in Malaysia including 18–46 deaths per year. Between 2008 and 2017, however, indigenous malaria cases decreased from 6071 to 85 (a 98.6% reduction), while *P. knowlesi* cases increased from 376 to 3614 cases.

Imported cases peaked in 2001 with a nadir in 2009 (Fig. 1). Although there are variable rates of imported cases throughout the study period, a decreasing trend has been observed. The imported cases were mostly from Papua New Guinea and Indonesia, with few cases from Nigeria and Pakistan.

Introduced cases were first identified in 2008 when they accounted for 78 cases (Fig. 1). Their number peaked in 2011 with 119 cases and then decreased to only 33 cases in 2012. In 2017, no introduced cases were detected; however, in 2018, 21 cases were detected. Overall, in 2018, there were a total of 4630 malaria cases reported in Malaysia, of which 4131 cases were *P. knowlesi* and 499 human malaria. The *Plasmodium* species responsible for human malaria were in the following order: *P. vivax* (284), *P. falciparum* (182), *P. ovale* (14), *P. malariae* (13), and mixed infection [6].

**Fig. 3:**
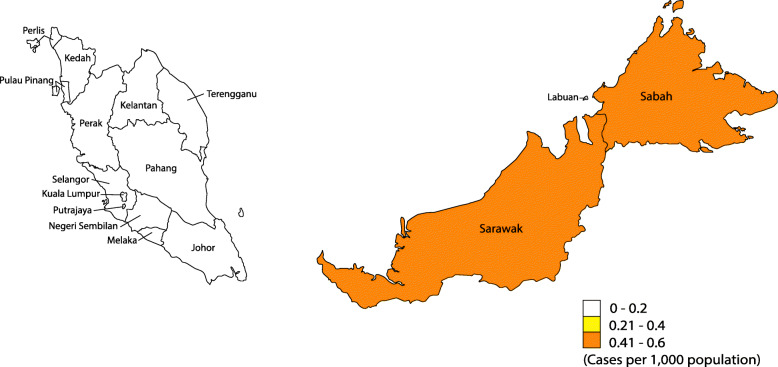
Distribution of *Plasmodium knowlesi* malaria cases by state in Malaysia, 2018

As illustrated in Fig. 3, zoonotic malaria cases, only refers to *P. knowlesi* cases in this manuscript, in Malaysia are not restricted to Sabah and Sarawak, two states located on the island of Borneo, where a significant proportion of the population is at risk of the disease, but also occurs in states with high forest coverage in Peninsular Malaysia, such as Kelantan, Perak, and Pahang [16].

**Fig. 4:**
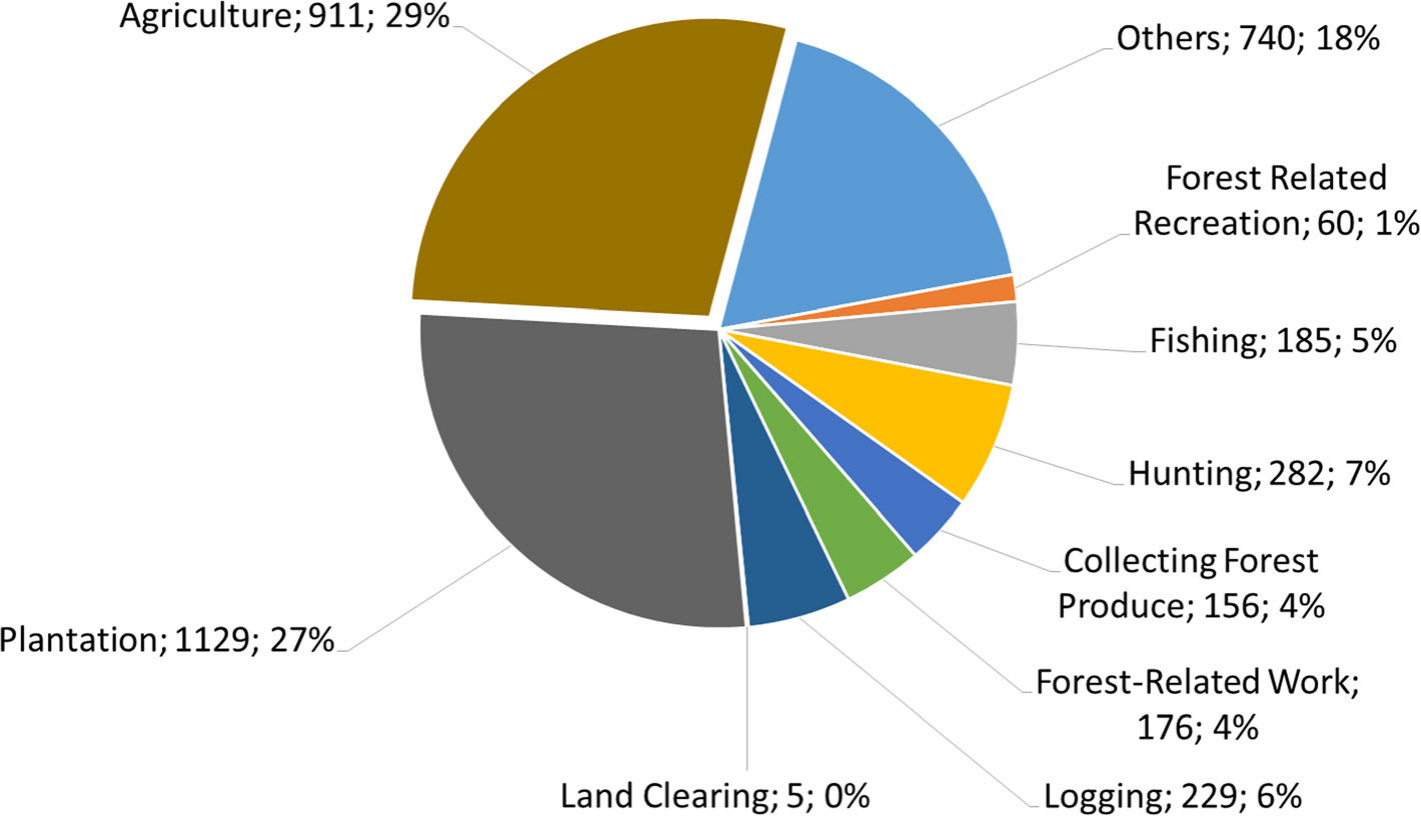
Distribution of *Plasmodium knowlesi* malaria by risk activities, Malaysia, 2018. Others are people in the dependent group such as housewife, student, and elderly

Figure 4 illustrates the distribution of zoonotic malaria according to risk activities in Malaysia in 2018. More than 50% of cases were in individuals involved with agriculture and plantation activities, with a large remainder proportion occurring in people engaged in forest-related activities.

Figure 2 illustrates the annual reported incidence trend for *knowlesi* malaria in Malaysia from 2008 to 2018. With current emphasis given by the Ministry of Health Malaysia on mandating hospital referral and admission policies for all malaria cases in both public and private sectors, mandatory referral for PCR testing of all microscopy-positive blood to be conducted by designated Public Health Laboratory, and surveillance linked to both systems, it is likely that the significant majority of symptomatic malaria cases were identified. Overall, *P. knowlesi* cases and incidence rates are increasing, with the highest reported case number of 4131 and the highest incidence rate of 0.13 case per 1000 population occurring in 2018.

## Discussion

A Malaria Eradication Program was launched in Malaysia in 1961 when there were recorded 243,870 cases per year throughout the country. However, it was not until the 1980s, following a renewed emphasis on malaria control by the Health Ministry, that a significant reduction in malaria cases was achieved. This resulted in a 6-fold reduction of reported malaria cases (44,226 cases) in 1980. Under Malaria Eradication Program and Malaria Control Program in Malaysia, vector control was performed by ensuring that drains and open pools of water (including seepage rainwater) be regularly and routinely sprayed with oil, large unused pools drained, residual spraying of homes with insecticide, and the use of mosquito net. Following further strenuous efforts over the subsequent 30 years, a further 7-fold reduction of malaria cases was achieved, and Malaysia entered the elimination phase in 2011. Malaria elimination is defined by the WHO as the interruption of local transmission (reduction to zero incidence of indigenous case) of a specified (human) malaria parasite in a defined geographical area as a result of deliberate activities. Malaysia achieved zero case of indigenous malaria in 2018, 2 years ahead of the scheduled target of 2020. Therefore, 2019 and 2020 are crucial for achieving malaria elimination status.

From 2008, PCR*-*based malaria diagnosis for *P. knowlesi* was initiated by Public Health Laboratory. Subsequently, significant increases in the incidence of this zoonotic parasite were observed to date. This could be due to various factors including improved diagnostic capacity, the reduction in human malaria cases, and a change in land use patterns creating increased opportunities for spillover of infections to humans through closer associations with natural reservoir hosts or access to infected vectors [17]. The successful control of human malaria in Malaysia may increase the susceptibility of populations to *P. knowlesi* due to the removal of cross-protective immunity. However, further research is needed to explain this phenomenon as a possibility.

The paradox for Malaysia is that despite the excellent efforts to control human malaria, and the elimination of these parasites from the country, malaria cases are rising due to increasing *P. knowlesi* transmission to humans. It is possible that Malaysia will achieve “elimination” of malaria despite higher malaria case numbers in the last 2 years than in 2014. *Plasmodium knowlesi* is classified as a zoonotic parasite as there is no evidence to date that human-to-human transmission can occur under natural conditions. Should such evidence arise, then *P. knowlesi* may be re-classified as human malaria, and Malaysia’s “elimination” status would need to be revised accordingly. Malaysia also faces the threat of imported malaria and the reintroduction of malaria due to the number of undocumented migrant workers traveling from endemic regions [18]. The number of imported malaria cases is further confounded by Malaysians returning from work in the agricultural and logging sectors of endemic countries [4].

Accurate identification of *P. knowlesi* is critical to the design and implementation of effective malaria interventions. Due to morphological similarities at the trophozoite stages, *P*. *knowlesi* is often mistaken for *P. malariae* by microscopy. It may also be mistaken for *P. falciparum* or *P. malariae* due to similar morphological features at some stages [19]. Thus, in the past, *P. knowlesi* may have been underdiagnosed, and its true incidence was likely considerably underestimated. For this reason, the definitive diagnosis of *P. knowlesi* is now carried out by PCR in Malaysia.

There are reports suggesting a lack of reliability among commonly used rapid diagnostic tests for the detection of *P. knowlesi* infections. Pan-plasmodium Rapid Diagnostic Tests (RDTs) can be used for screening (they will give a positive result for malaria parasites) but not for species confirmation of *P. knowlesi*. Detection of *P. knowlesi* using RDTs has demonstrated low sensitivity [20]. PCR, on the other hand, can detect and diagnose *P. knowlesi* with high sensitivity and specificity; however, it is expensive and requires specialized equipment and highly trained personnel [21].

Loop-mediated isothermal amplification is, potentially, a reliable and convenient substitute for the molecular diagnosis of *Plasmodium* infection particularly in high malaria transmission regions as it displays high sensitivity and specificity as well as its low cost and high simplicity, making it particularly useful as a point-of-care diagnosis [22]. Other methods such as recombinase polymerase amplification (RPA) also have the potential to be developed as point-of-care molecular diagnostic tools for *P. knowlesi* infections [23]. Poor diagnostics contribute to inadequate surveillance for *P. knowlesi*, and calls have been made to include specific *P. knowlesi* reporting to the WHO to encourage improved regional surveillance [24].

**Fig. 5:**
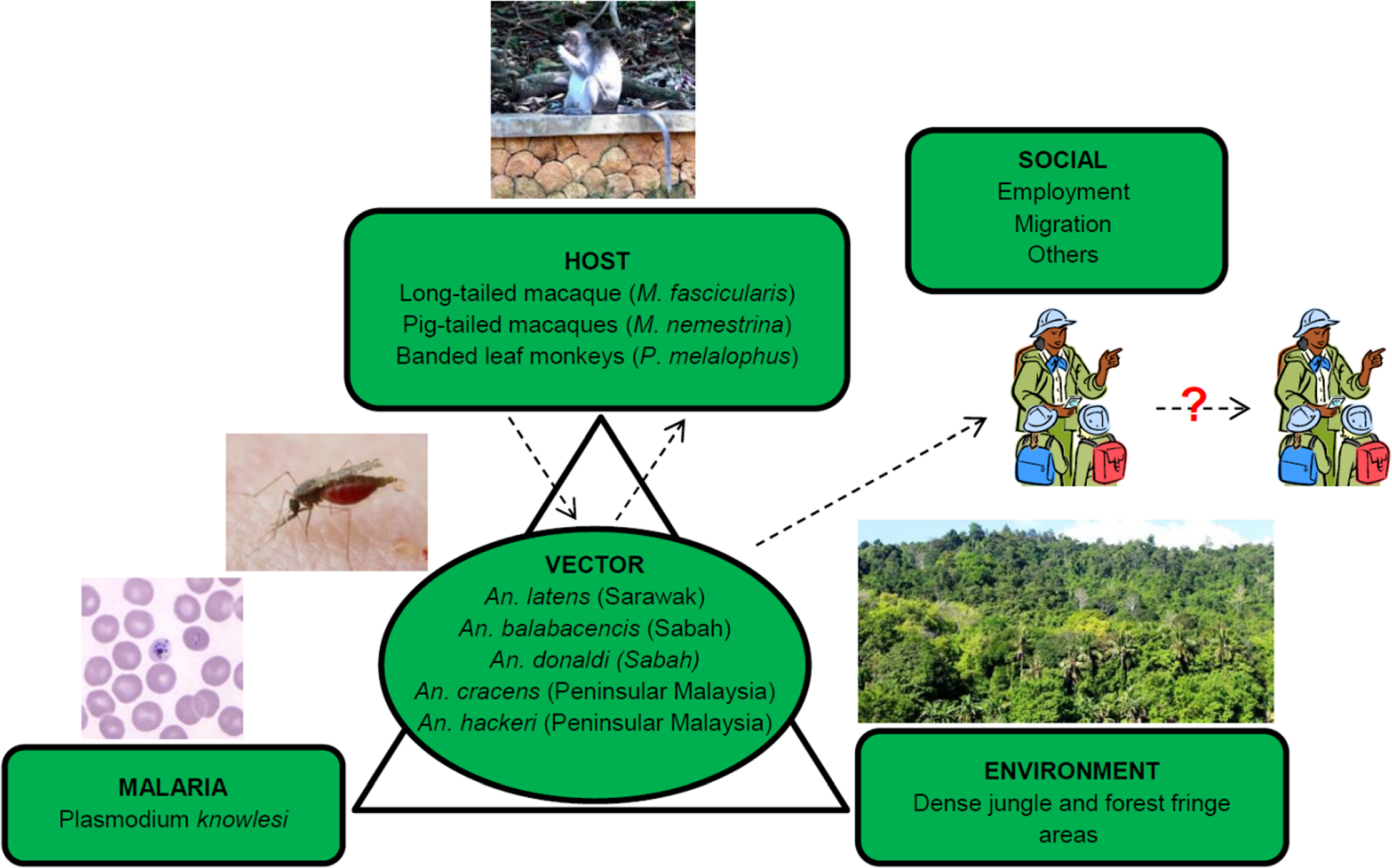
Epidemiologic triad and factors for zoonotic malaria infections

Figure 5 describes the epidemiologic triad of zoonotic malaria infections. *Plasmodium knowlesi* cases are proportionally higher in states with higher coverage of intact forest as opposed to forest loss areas [16]. Vector mosquitoes, members of the *An. leucosphyrus* group, are found throughout forested regions of Malaysia and are particularly associated with dense jungle and forest fringes. *Anopheles leucosphyrus* mosquitoes are exophagic, typically feeding and resting outdoors after dusk. In Sarawak, the forest-breeding *An. latens* was found to be the primary vector of *P. knowlesi* [25]. *Anopheles latens* has also been found to harbor other simian malaria parasites including *Plasmodium inui*, *Plasmodium coatneyi*, and *Plasmodium fieldi*. This species mainly breeds in forests in locations comprised of shaded temporary pools and natural containers on the ground [26].

*Anopheles balabacensis* has been implicated as a vector of *P. knowlesi* in Sabah. This species prefers to breed in ground pools formed in, for example, fruit orchards and rubber and palm oil plantations [27]. In Peninsular Malaysia, the major *P. knowlesi* vector is thought to be *Anopheles cracens* [26]. However, knowledge regarding the distribution of these vectors in the country is sparse [28].

Risk-assessment maps for *P. knowlesi* have been produced based upon the ranges of the host species, vectors, land use patterns, and confirmed infection of humans, macaques, and mosquitos [29]. *Plasmodium knowlesi* transmission to humans peaks in June [30], and this is associated with peak average monthly rainfall and humidity with a lag time of 3 months [31].

**Fig. 6:**
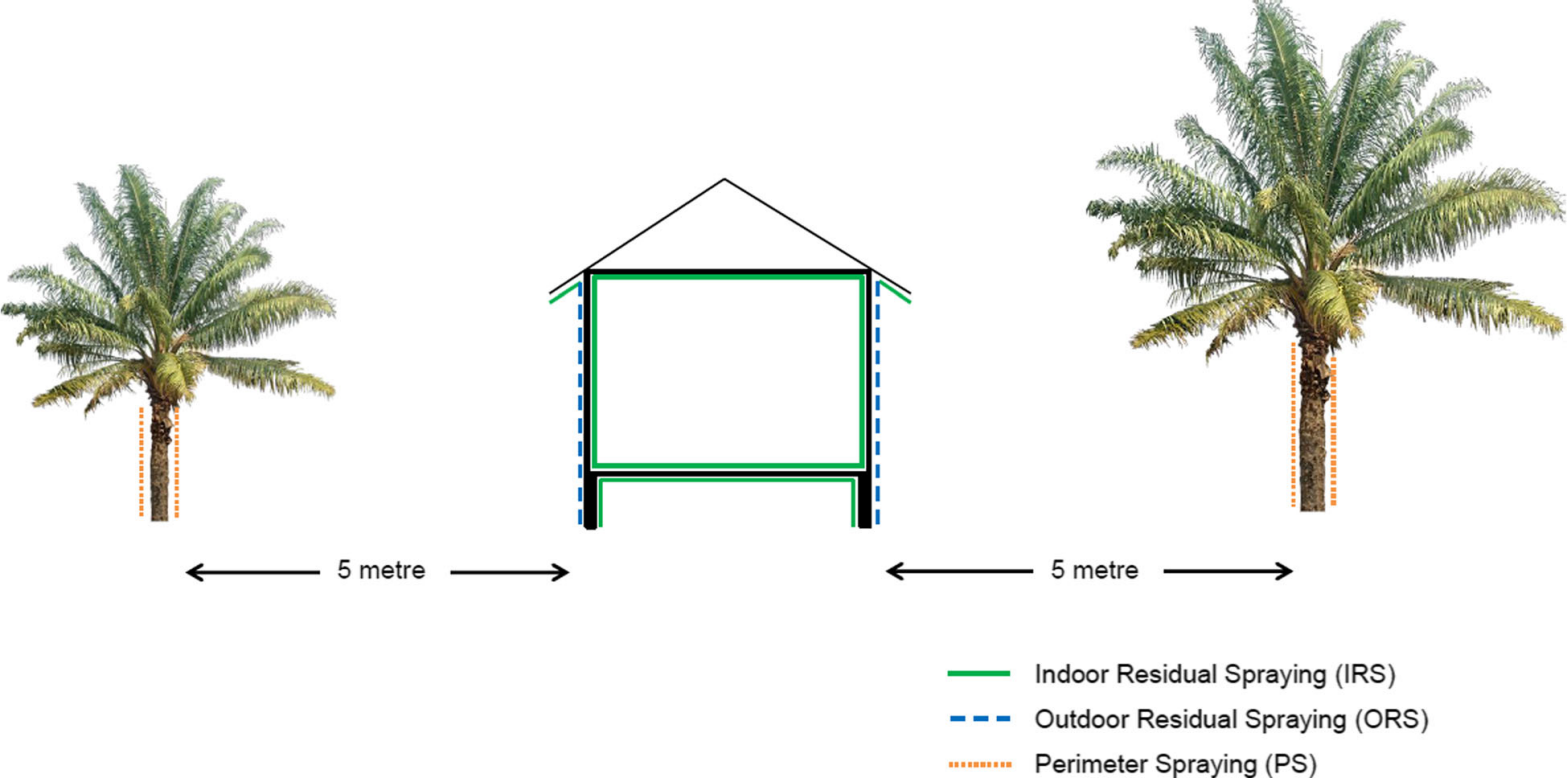
Schematic diagram of insecticidal sprays used for vector control

Vector control methods for *knowlesi* malaria include indoor residual spraying (IRS), and experimental methods of outdoor residual spraying (ORS), and perimeter spraying (Fig. 6). Outdoor residual spraying is done to the outside walls of a house. Perimeter spraying on the other hand is conducted up to the height of trunks for all trees in the range of less than 5 m from a house (where adult vectors are predominantly found resting). Based on a study conducted by Malaysia’s Institute of Medical Research (IMR) in Tenom, Sabah, a polymer-enhanced suspension concentrate (SC-PE) synthetic pyrethroid dose of 25 mg/m^2^ is effective to maintain 80% mortality of *An*. *balabacensis* for at least 6 months when applied to timber, bamboo, and concrete [32].

Control and prevention of zoonotic malaria remains a major challenge especially in rural areas, where macaques are common [33]. Macaque populations tend to congregate near village areas (average 6 km away) as well as within evergreen and deciduous forest cover, and are associated with lower elevations and higher temperatures [34]. Reducing macaque population density would be an effective means of controlling *P. knowlesi* transmission, but implementing such a measure is impractical and difficult as well as unethical.

The highest incidences of zoonotic malaria occur in the states of Sabah, Sarawak, and the Gua Musang district of Kelantan state [35]. In an attempt to identify the environmental risk factors for *P. knowlesi* in Sabah, it was found that agricultural expansion and forest fragmentation positively affect *P. knowlesi* exposure, supporting linkage between land use change and *P. knowlesi* transmission [36]. Factors that bring human populations into closer and more frequent contact with macaque monkeys exacerbate zoonotic transmission of pathogens, including malaria, from non-human primates to man. Land-use change also affects macaque habitat use and ranging behavior, with repercussions for changes in human disease risk [37]. Indonesia and Malaysia have increased oil palm cultivation from 2.6 million ha in 1990 to over 15 million ha by 2014, leading to large scale deforestation [38].

Men working in agricultural areas have the highest risk of *P. knowlesi* infection [39, 40], with occupations in industries such as forestry, agriculture, and hunting identified as risk factors [39, 41, 42]. In Sabah, *P. knowlesi* infections were recorded affecting a range of ethnic groups, with Rungus, Dusun, and Murut accounting for 32%, 28%, and 15% of infections, respectively [43]. This may be related to their residence in forest fringe areas and the fact that their livelihoods are primarily based on forest-related activities.

The prevention and control of *knowlesi* malaria in rural settings relies primarily on vector control, most importantly the use of personal anti-mosquito measures when working in forest-fringe areas. This involves the use of insect repellents, but these are prohibitively expensive for subsistence farmers, hunters, logging camp workers, and other rural people whose daily activities take them to the forest and forest fringes. Other, more novel interventions could be considered, for example, the use of insecticide impregnated hammocks which have been successful for controlling forest malaria in Vietnam [44]. However, although some people stay overnight in the forest in Malaysian Borneo, hammocks are not traditionally used here, and so such a control measure would be difficult to implement successfully.

There is an urgent need to foster inter-sectoral collaboration for the control of *knowlesi* malaria. Additional efforts by the Ministry of Health may include the strengthening of health education and health promotion by local health authorities as well as the strengthening of the currently existing community workforce, *Sukarelawan Penjagaan Kesihatan Am* (*SPKA*) also known as General Healthcare Volunteer, and increasing rural community resilience. The Wildlife Department could undertake the role of screening macaques for malaria parasites, controlling their numbers, and may also assist the Ministry of Health in the provision of health education for hunters. The Forestry Department could monitor forest activities in view of infection prevention such as keeping full record of people going in and out of forest and advocating use of mosquito bite prevention methods. The Malaysian Armed Forces could conduct screening of staff as well as the supervision and monitoring of the uptake of chemoprophylaxis. Due to the location of camps and activities involving forest areas, this group is deemed to be of high risk for *P. knowlesi* malaria infection [45]. For the plantation sector, key players can be involved by appointment of a “Malaria Ambassador” who will collaborate with the State Health Department in malaria screening and also ensure provision of insecticide-treated bed nets to workers to prevent outdoor biting as many people prefer to sleep outdoor during hot weather.

Additional research on mapping of vector breeding site, determining prevalence of malaria parasite infection, identifying and tracking areas of macaque population, and stricter policy on planning of land opening will aid in eliminating zoonotic malaria.

## Conclusion

The changing epidemiology of malaria in Malaysia from indigenous malaria to zoonotic malaria has had a severe negative impact on the gains made by more than half a century’s effort by the Malaysian government in the battle against the disease. More research is required in order to effectively combat and eliminate *P. knowlesi* in humans. Improved inter-sectoral collaboration is required in order to develop a more effective, integrated effort that involves all stakeholders. Local political commitment and provision of technical support from the World Health Organization can help to create focused and concerted efforts towards ensuring the success of the National Malaria Elimination Strategic Plan.

## Data Availability

The datasets used and/or analyzed during the current study are available from JJ, Disease Control Division, Ministry of Health, Putrajaya, Selangor, Malaysia, on reasonable request.

## Abbreviations

CI: Confidence interval
PCR: Polymerase chain reaction
CFR: Case fatality rate
SPKA: *Sukarelawan Penjagaan Kesihatan Am*/General Healthcare Volunteer
IRS: Indoor residual spraying
ORS: Outdoor residual spraying
IMR: Institute of Medical Research
SC-PE: Suspension Concentrate Synthetic Pyrethroid
